# Concurrent and Longitudinal Relationships between Positive Youth Development Attributes and Adolescent Internet Addiction Symptoms in Chinese Mainland High School Students

**DOI:** 10.3390/ijerph18041937

**Published:** 2021-02-17

**Authors:** Diya Dou, Daniel T. L. Shek

**Affiliations:** Department of Applied Social Sciences, The Hong Kong Polytechnic University, Hong Kong, China; diya.dou@polyu.edu.hk

**Keywords:** positive youth development, life satisfaction, Internet addiction, China, secondary students

## Abstract

In view of growing adolescent Internet addiction (IA) in the global context, there is a great need to understand the predictors of IA and design related evidence-based intervention and prevention programs. This longitudinal study investigated the relationships between Positive Youth Development (PYD) attributes and IA problems and the mediating role of life satisfaction using a large sample of Chinese high school students (*N* = 2648). Separated by one academic year, students completed a questionnaire evaluating their adolescent psychosocial adjustment, including validated PYD and IA measures. Multiple regression and structural equation modeling analyses were used. Consistent with the theoretical predictions of PYD models, results revealed a significant negative influence of PYD attributes on IA symptoms concurrently and longitudinally. In particular, general PYD attributes, such as emotional competence, spirituality, and resilience, showed strong and stable protective effects against IA. Life satisfaction also served as a mediator of the influence of all measures of PYD attributes on IA symptoms. The study underlines the importance of PYD attributes in promoting adolescents’ life satisfaction and preventing IA, and thus contributes to the design and implementation of evidence-based intervention and prevention programs.

## 1. Introduction

The Internet is widely used in business and household sectors in contemporary society. The number of Internet users in China reached 940 million by June 2020, and among them were 172 million youths under 19 years [1]. The top five online activities that Chinese youths frequently engaged in included online learning, listening to music, playing video games, online chatting, and watching short videos [1].

Although the Internet is beneficial for adolescents in accessing information and building social relationships, Internet addiction (IA) among adolescents has become a serious social and mental health issue. IA is defined as a “maladaptive pattern of Internet use leading to clinically significant impairment or distress” [2]. It is manifested as pathological Internet use, which often leads to impairments in academic, occupational, and social functioning [3]. Adolescents are particularly at high risk of IA due to significant neurobiological, social, and cognitive changes taking place in the developmental course of adolescence [4]. IA symptoms often coexist with other developmental difficulties and health problems. For example, excessive Internet use is found to be adversely associated with adolescents’ academic performance [5]. Primarily, students with IA often experience distortion in time perception when engaging in online activities and thus leave less time for academic work. Besides, IA also impairs adolescents’ cognitive abilities [2], such as the capability to concentrate [6]. IA is also associated with physical health issues, such as back pain and strain injury. Moreover, IA often coexists with other internalizing and externalizing problems, such as depression, hostility, anxiety, self-harm behaviors, and substance abuse [7,8,9]. The recent research of Ruggieri et al. revealed a close relationship between problematic social media use and social anxiety among adolescents and their mothers, demonstrating the key role of family in intergenerational transmission of problematic Internet use [10]. In addition, IA may hinder adolescents from building positive social functioning in the real world [11,12]. The prevalence rate of IA among Chinese youths has reached 10% in recent years [13], implying that some 17 million youths are suffering from IA. Therefore, identifying critical predictors of IA and its underlying psychological mechanisms are essential for designing evidence-based intervention and prevention programs.

Previous adolescent research has often followed a deficit model of youth development, which upholds the belief of the inevitability of “storm and stress” in adolescence. Therefore, much effort has been devoted to providing effective treatment and crisis intervention for adolescents with mental and behavioral disorders such as IA [7,14]. While schools and education bureaus promote school-based interventions and counseling services [15], governments formulate policy responses to address adolescent IA. For example, the anti-addiction system for major online-gaming sites was launched in China in 2007 and was further expanded to video-streaming platforms in 2019 [1].

Empirical evidence has increasingly shown that adolescents can demonstrate tremendous potential and strength during this developmental stage, which is inconsistent with the deficit model [16]. Instead of solely focusing on crisis intervention and treatment for problem behavior, psychologists have begun examining protective and risk-reducing factors in adolescent development to promote subjective well-being [17]. Previous studies have revealed that adolescents with limited psychological and environmental resources, such as self-efficacy, self-control, and social support, are vulnerable to developmental problems [18,19]. Positive youth development (PYD) has, in recent years, emerged as a coherent approach offering a strength-based and positive model of youth for health and well-being promotion [17,20]. However, very few researchers have used the PYD perspective and related validated scales to investigate the association between PYD attributes and adolescent IA [21]. Besides, the mechanisms underlying the protective effects exerted by PYD attributes on adolescents remain unclear. Life satisfaction is theoretically considered a key mediating factor in influencing adolescent development but remains under-researched on the IA issue [22,23,24]. This study examined the predictive effects of PYD attributes on adolescent IA and the mediating effect of life satisfaction using two waves of longitudinal data. 

### 1.1. Positive Youth Development Attributes and IA

The PYD approach envisions youths as resources rather than as problems. It highlights the importance of adolescents’ potentials, strengths, preferences, and the interactions between individuals and environments [20]. In the mid-90s, Benson [25] proposed a framework of “developmental assets” in adolescent development, which forms a solid basis of the PYD approach. Benson identified forty external and internal developmental assets that matter to youth health and development. Particularly, internal assets include a set of positive personal characteristics, such as positive values, social skills, and positive identity. Lerner [26] conceptualized the “Five Cs” model of PYD, including “Connection,” “Confidence,” “Competence,” “Character,” and “Caring.” Based on a thorough review of PYD programs worldwide, Catalano [27] identified 15 PYD attributes that were commonly emphasized in effective PYD programs. Empirical studies have shown that possessing different PYD assets, including cognitive-behavioral competence, prosocial attribute, and positive identity would help prevent various problematic behaviors such as IA [28,29,30,31]. Shek [32] also argued that the PYD approach is a promising intervention strategy to respond to adolescent gaming problems.

Regarding cognitive and behavioral competence in the PYD framework, this refers to thinking logically, making hypotheses, exploring possibilities, making rational decisions, and performing appropriate behaviors accordingly. As IA is often regarded as a behavioral disorder, cognitive behavior approach therapy has received the most empirical investigation in practice among different therapies for IA intervention [33]. If adolescents possess strong cognitive thinking skills and behavioral competence, they can identify their needs met by the Internet, the negative impact of IA, and spontaneously regulate their Internet use. According to Deci and Ryan’s self-determination theory [34], strong self-determination enables adolescents to integrate regulation and intrinsic motivation and thus would promote their healthy Internet use. For example, based on a survey conducted with 694 Chinese adolescents, Li et al. [35] found that higher levels of self-control were associated with decreased IA symptoms. Similarly, Mills and Allen found that autonomous self-regulation was negatively correlated with IA and positively correlated with adaptive motivations [36].

Adolescent identity as a component of PYD has also been linked to Internet use in the literature. On the one hand, the Internet could be a valuable channel for adolescents to explore their identity in modern societies [37]. As the Internet provides an environment for anonymous social interaction, adolescents with self-identity confusion may seek advice online from peers [38]. On the other hand, some adolescents with self-identity confusion tend to over-use the Internet as a coping strategy. Rodger and Melioli [39] have argued that inappropriate Internet use often coexists with adolescent over-concerns about body images and eating disorders. Dumas et al. [40] revealed that identity-achieved adolescents engaged in less risky behaviors than those with a diffused identity. Similarly, Agbaria and Bdier’s research [41] also showed that adolescents with a clear identity tended to show fewer IA symptoms. Generally speaking, research evidence has supported the idea that clear self-concepts and positive identity have a negative association with IA.

Prosocial attributes are another dimension of PYD which has been found to play an important role in regulating Internet use among adolescents [42]. Prosocial attributes concern the intention to benefit others and follow social norms and regulations. People with prosocial attributes tend to demonstrate more socially desirable behaviors and less socially undesirable behaviors [43]. According to social control theory, adolescents with strong bonding with prosocial peers and adults are less likely to be involved in delinquent behaviors [44]. Empirical research has revealed a protective role of prosocial peer affiliation in preventing IA and buffering the negative impact of cyberbullying among adolescents [45]. Other studies have also shown that adolescents with IA issues tended to show fewer prosocial behaviors [42]. However, some researchers also argued that prosocial and helping behavior might cost extra psychological resources and place a moral burden on the self, which may harm individual well-being [46].

Finally, other general PYD attributes, such as emotional competence, social skills, resilience, and spirituality, are also important personal assets for adolescents. As IA often serves as an escape from difficulties and temporary relief from negative emotions, these attributes prevent IA by allowing adolescents to deal with negative emotions, bounce back from adverse life events, and pursue long-term personal goals [20]. Blasi et al. [47] argued that, for individuals who experienced difficulties in regulating adverse emotions, online gaming was more likely to become a coping strategy. Similarly, adolescents who lack social skills are likely to develop IA [48]. Generally speaking, research has shown that possessing strong emotional competence, resilience, and spirituality would allow adolescents to adopt active coping strategies instead of escape-avoidance coping, which is often associated with IA. Shek [32] also argued that general PYD attributes should be promoted as protective factors for adolescent Internet gaming problems.

### 1.2. The Mediating Role of Life Satisfaction

Although studies have demonstrated a negative relationship between different PYD attributes and adolescent problem behavior such as IA [28,29,30,31], the related psychological mechanisms are less clear. Particularly, although life satisfaction is theoretically considered a key mediating factor in the process of adolescent development [22,23,24], its role in mediating the relationship between PYD attributes and IA is under-researched.

According to the strength-based approach, personal strengths are vital levers for life satisfaction and happiness [49]. These strengths help adolescents cope with stressful events, establish positive social relationships, maintain healthy lifestyles, and subsequently promote psychological well-being [50]. Empirical studies also support these theoretical arguments. For example, self-determination reflects one’s intrinsic motivation, which is an important predictor of life satisfaction [51]. It was also found to buffer the negative influence of stressful life events on life satisfaction [52]. Prosocial behavior was also positively related to life satisfaction because applying prosocial behavior brings positive and pleasant experiences, which in turn contributes to adolescents’ life satisfaction [53]. In addition, adolescents who built their identity on true self put more effort into self-care and goal pursuit, which often increase positive life experiences [54]. Moreover, adolescents who developed a higher level of resilience tend to explore more resources in order to live better, and thus feel more satisfied with their life [55].

A large body of literature has demonstrated that life satisfaction is closely associated with mental health problems. Based on a longitudinal cohort study involving 1265 participants, Fergusson et al. [56] found that people with high levels of life satisfaction demonstrated less substance dependence, depression, anxiety disorder, and suicidality. Similar results were found in Asian countries [57] including Chinese societies [31]. The relationship between life satisfaction and IA has gained more attention in recent years. For example, Cao et al. [58] revealed a negative association between adolescent problematic Internet use and all dimensions of life satisfaction. Another study involving 1552 adolescents reported similar findings that the severity of problematic Internet use was typically associated with well-being measures [59]. As IA is often regarded as a maladaptive response to life difficulties and stressful events, adolescents who are dissatisfied with their lives in the real world tend to escape from reality and indulge in the virtual world for a long time. In contrast, when people feel satisfied with their lives, they tend to use positive and active coping strategies and are less likely to be engaged in addictive behaviors [60,61]. Although existing research has consistently revealed negative associations between life satisfaction and health problems, research on adolescent IA and psychological well-being are still limited [58].

Several research gaps are identified in the existing scientific literature. First, very few studies have specifically explored the role of life satisfaction in mediating the relationship between positive attributes and adolescent problem behaviors. Although one recent study revealed that life satisfaction mediated the relationship between PYD attributes and Chinese adolescent delinquency [31], there is no systematic research in mainland China. Another research gap concerns the inconclusive results on the relationships between specific PYD attributes and adolescent well-being. For example, the study of Amdurer et al. [62] showed that systemic thinking demonstrated a negative effect on life satisfaction. Similarly, evidence on the relationship between prosocial attribute and IA seems less definitive as compared to that based on cognitive-behavioral competence [63]. Hence, the present study responded to the research gaps by investigating the influence of PYD attributes and life satisfaction on IA based on a two-wave longitudinal study.

### 1.3. Sociodemographic Factors and IA

Based on the literature, we controlled three sociodemographic factors in the present study, including gender, age, and family intactness.

Adolescent boys are generally hypothesized to be more vulnerable to addiction. Several studies reported positive relationship between being male and IA [64]. Based on a sample of 1618 Chinese high school students, the research of Lam et al. [65] revealed that the risk of being addicted to the Internet for boys was 50% higher than that for girls. Similar results were found in a three-year longitudinal study conducted with Hong Kong adolescents [66]. One possible explanation is that men are more likely to engage in online gaming and gambling [67]. However, some studies demonstrated non-significant gender differences in adolescent IA [68]. Moreover, recent research revealed that women are more prone to smartphone addiction than men, possibly due to more social networking and messaging [69]. Therefore, we included gender as a control variable in the present study.

Age is also found as a key risk factor of IA [70]. Compared to adults, adolescents showed higher levels of IA. In a similar vein, the study of Blasi et al. [47] also found that younger students demonstrated more IA symptoms related to online video games. In Shek and Yu’s six-wave longitudinal study [30], both boys and girls generally reported a decreased frequency of IA behavior across the six waves. The only exception was that students in Grade 8 reported a slight increase in IA compared with those in Grade 7. It is possible that adolescents may increase their Internet use due to stronger needs for autonomy, independence, and social interaction when entering secondary school education [71], but adolescent risk behavior such as IA would generally fade out over time [30].

Family intactness is an important factor related to adolescent IA. A nonintact family environment is an adverse condition for adolescent development. Previous research has revealed that adolescents living in non-intact families tended to develop more risk behaviors than their counterparts [72]. Adolescents living in intact families showed less IA symptoms such as excessive use and withdrawal symptoms [30]. It is argued that nonintact family may provide insufficient parental monitoring and create additional emotional stress for children, in either way increasing the risk of IA [73]. Empirically, Shek and Yu [30] found that adolescents in nonintact families in Hong Kong were more likely to demonstrate IA behaviors than their counterparts.

### 1.4. Research Questions

Based on data collected from Chinese high school students over two years, we attempted to answer the following research questions in this study.

*Research Question 1 (RQ1)*: What is the relationship between PYD attributes and IA?

Based on the previous findings [66,74], we proposed that PYD attributes would be negatively associated with IA concurrently (Hypothesis 1a) and longitudinally (Hypothesis 1b).

*Research Question 2 (RQ2)*: What is the relationship between PYD attributes and life satisfaction?

With reference to the previous studies [75,76], we proposed that PYD attributes would be positively associated with life satisfaction concurrently (Hypothesis 2a) and longitudinally (Hypothesis 2b).

*Research Question 3 (RQ3)*: What is the relationship between life satisfaction and IA?

In line with the previous studies [77], it was hypothesized that life satisfaction would negatively predict IA concurrently (Hypothesis 3a) and longitudinally (Hypothesis 3b).

*Research Question 4 (RQ4)*: Does life satisfaction mediate the effect of PYD attributes on IA?

Based on the previous studies suggesting the mediating role of life satisfaction [22,31,78,79], we hypothesized that life satisfaction would mediate the prediction of PYD attributes on IA (Hypothesis 4).

## 2. Methods

### 2.1. Participants and Procedures

The data were collected from students in Grade 7 and 8 in four high schools in Guangdong, Jiangsu, and Jiangxi provinces in mainland China by employing convenience sampling. The four schools were all public schools with class sizes ranging from 27 to 58 students. The first data collection exercise took place at the beginning of the school year of 2016/2017. All students in Grade 7 and 8 in the four schools were invited to respond to a questionnaire on adolescent development and well-being using a paper-and-pencil method. Before the data collection, consent was obtained from school administrators, teachers, participating students, and their parents. The data were collected in the classroom during school hours. Trained teachers administered the survey and explained to students the research purposes and important principles, including the anonymity and confidentiality of data collection and use. This study had been reviewed and approved by the Human Subjects Ethics Sub-committee (HSESC) of The Hong Kong Polytechnic University.

In total, 1362 Grade 7 students and 1648 Grade 8 students participated in the survey at Wave 1. The data collection of the second wave was conducted one year later. A total of 1305 Grade 8 students and 1343 Grade 9 students completed the same questionnaire at Wave 2. The attrition rates were 4.19% for Grade 7 students and 18.51% for Grade 8 students. The matched sample included 2648 students (*M_age_* = 13.12 years). Among them, 57.1% were boys (*N* = 1513), 41.9% were girls (*N* = 1109), and 1.0% did not report their gender (*N* = 26). As to the family characteristics, 84% were from intact families (*N* = 2225) and 15.1% reported that their parents were divorced or separated (*N* = 401). In total, 24.4% students (*N* = 645) were from one-child families and 73.7% reported that they had brothers or sisters (*N* = 1951).

An attrition analysis was conducted to examine whether adolescents who participated in both waves (*N* = 2648) differed from the dropouts (*N* = 362). No significant differences were found for age, gender, family intactness, and IA in both grades. The dropouts reported higher scores on some subscales and total PYD measure than did the non-dropouts in the same grade (Mean difference ranged between 0.13 and 0.36, *t* ranged from 2.28 to 3.80, *p*s < 0.05, and Cohen’s *d* ranged between 0.16 and 0.36). The dropouts in Grade 8 reported higher levels of life satisfaction (Mean = 4.30) than their counterparts (Mean = 3.98; Mean differences = 0.31, *t* = 4.59, *p* < 0.001, Cohen’s *d* = 0.29). As the effects are considered relatively insignificant [80], the attrition bias is not a major concern of the present study.

### 2.2. Measures

The questionnaire included several measures examining various aspects of adolescent development and well-being. In this study, we mainly focused on students’ PYD attributes, life satisfaction, and IA.

*PYD attributes.* An 80-item “Chinese Positive Youth Development Scale (CPYDS)” was used to measure students’ PYD attributes. CPYDS has been widely used in research examining PYD attributes of different Chinese adolescent population groups, including junior and senior secondary school students in Mainland China and Hong Kong [81,82]. Previous research has revealed that CPYDS is a validated measure possessing good psychometric properties [83,84]. CPYDS includes 15 subscales, which are in accordance with the 15 PYD attributes identified in Catalano’s review [27]. These 15 subscales are further categorized into four higher-order factors. The first factor is “Cognitive-behavioral competence” (CBC, e.g., “I know how to see things from different angles”), including “cognitive competence,” “behavioral competence,” and “self-determination” subscales. The second dimension is “Prosocial attribute” (PA, e.g., “I care about unfortunate people in society”), which contains “prosocial norms” and “prosocial involvement.” The third factor is “Positive identity” (PIT, e.g., “I am a person with self-confidence”), which consists of “clear and positive identity” and “beliefs in the future” subscales. The last higher-order factor is “General positive youth development” (GPYD, e.g., “When I am unhappy, I can appropriately show my emotions”), including subscales of “bonding,” “resilience,” “social competence,” “recognition for positive behavior,” “emotional competence,” “moral competence,” “self-efficacy,” and “spirituality.” A 6-point Likert scale was used (1 = “Strongly disagree” and 6 = “Strongly agree”). We used the mean scores of each higher-order factor in the analyses. Results of scale reliability analysis show that all higher-ordered PYD measures used in the present study possessed sufficient internal consistency (Cronbach’s *αs* ranged between 0.86 and 0.96, see Table 1). Although the measure of self-efficacy appeared to have a relatively “lower” alpha value, we kept it in the analysis as a primary PYD attribute for the following reasons. Firstly, we adopted an integrated conceptual framework including all 15 PYD attributes suggested by Catalano et al. [27]. Secondly, the data analyses were based on higher-order composite factors (e.g., general PYD attribute) rather than primary PYD attributes (e.g., self-efficacy). Thirdly, the values of the mean inter-item correlation at Wave 1 (0.37) and Wave 2 (0.42) were acceptable and some researchers suggest that a lower level of Cronbach’s alpha value (e.g., 0.54) can be considered acceptable for a scale with two items [85].

*Life satisfaction.* The “Satisfaction with Life Scale” was developed by Diener et al. [86]. We used a Chinese version translated by Shek [87], which showed good reliability and validity in previous studies examining life satisfaction among the Chinese population [88]. A sample item is “In most ways my life is close to my ideal.” A 6-point rating scale was used (1 = “Strongly disagree” and 6 = “Strongly agree”). A higher score indicates a higher level of life satisfaction. The internal consistency of the scale at both waves was good (Cronbach’s *αs* were 0.81 and 0.84 at Wave 1 and Wave 2, respectively, see Table 1).

*Internet addiction.* The Internet Addiction Scale developed by Young [2] was used to measure IA symptoms. The present study used a Chinese version, which has exhibited good psychometric properties in previous research conducted with Chinese adolescents [89]. Students responded to 10 items by indicating whether they had demonstrated the corresponding IA symptoms in the past year using a Yes/No dichotomous scale. A sample item is “Do you feel a need to spend more and more time online to achieve satisfaction?” A total score was calculated based on the counts of “yes” answers. The values of Cronbach’s α were 0.77 and 0.80 at Wave 1 and Wave 2, respectively (see Table 1).

### 2.3. Data Analyses

Descriptive statistics and Pearson correlation analyses for the research variables were performed using SPSS version 26.0. As discussed earlier, different PYD attributes may exert distinct effects on adolescent development. Therefore, separate analyses were carried out to examine the relationships between different measures of PYD attributes and other research variables.

For RQ1, correlation and multiple regression analyses were conducted to examine the concurrent and longitudinal prediction of each subscale and total PYD scores on IA. The same analyses were conducted to understand the prediction of PYD attributes on life satisfaction (RQ2) and the prediction of life satisfaction on IA (RQ3).

To further explore the role of life satisfaction in the relationship between PYD attributes and IA, we analyzed several mediation models using PROCESS macro in SPSS [90]. A series of regression analyses were performed to examine the mediating effect of life satisfaction on the relationship between each subscale and total PYD scores and IA (RQ4). Bootstrapped bias-corrected (BC) 95% confidence intervals (CIs) were calculated for regression coefficients using 2000 re-samplings in the mediation analyses [90].

As PROCESS can only deal with one PYD measure at one time, we further conducted structural equation modeling (SEM) analysis using the lavaan package [91] in the R software. By using SEM, both observable and latent variables could be accommodated in one model, allowing hypothesis testing of the relationship among PYD attributes, life satisfaction, and IA. The fit indices included the χ^2^, Comparative Fit Index (CFI), Tucker-Lewis Index (TLI), Non-Normed Fit Index (NNFI), Root Mean Square Error of Approximation (RMSEA), and Standardized Root Mean Residual (SRMR) to measure the model fit [92]. This approach was adopted in Zhu and Shek’s recent work [31].

## 3. Results

### 3.1. RQ1: Predictive Effects of PYD Attributes on IA

Table 2 summarizes the results of descriptive statistics and Pearson correlation coefficients of research variables. Compared to adolescent girls, boys tended to report higher levels of IA. Besides, younger adolescents tended to have lower levels of IA. Moreover, students from non-intact families tended to report higher levels of IA. Correlation analyses also suggested that all PYD measures were negatively associated with IA at each wave and over time (*r* ranged between −0.14 and −0.32, *p*s < 0.001).

Hierarchical multiple regression analyses further showed that after controlling age, gender, and family intactness, all PYD measures showed significant concurrent effects on IA at each wave (*β* ranged between −0.21 and −0.31, *p*s < 0.001, Cohen’s *f^2^* ranged between 0.045 and 0.106, see Table 3). Also, all Wave 1 PYD measures had significant longitudinal effects on Wave 2 IA (*β* ranged between −0.14 and −0.20, *p*s < 0.001, Cohen’s *f^2^* ranged between 0.018 and 0.040, see Table 4). Furthermore, after controlling Wave 1 IA, results showed that all Wave 1 PYD measures predicted a decrease in IA over time (*β* ranged between −0.04 to −0.07, *p*s < 0.05, Cohen’s *f^2^* ranged between 0.002 to 0.005, see Table 4).

### 3.2. RQ2: Predictive Effects of PYD Attributes on LS

Results of correlation analysis revealed significant positive correlations between different subscales and total PYD scores and life satisfaction at each wave (*r* ranged between 0.46 and 0.61 and between 0.44 and 0.58 at Wave 1 and Wave 2, respectively, *p*s < 0.01, see Table 1). Among different subscales of PYD, general PYD attributes and positive identity showed the strongest correlations with life satisfaction. Results of multiple regression analyses demonstrated that different subscales and total PYD had significantly positive effects on life satisfaction at each wave (*β* ranged between 0.44 and 0.51, *p*s < 0.001, Cohen’s *f^2^* ranged between 0.234 and 0.587, see Table 5). In addition, all Wave 1 PYD measures significantly predicted Wave 2 life satisfaction (*β* ranged between 0.18 and 0.27, *p*s < 0.001, Cohen’s *f^2^* ranged between 0.033 and 0.079, see Table 6). After controlling Wave 1 life satisfaction, positive identity, general PYD, and total PYD significantly predicted an increase in life satisfaction (*β* ranged between 0.05 and 0.07, *p*s < 0.05, Cohen’s *f^2^* ranged between 0.002 and 0.003, see Table 6).

### 3.3. RQ3: Predictive Effects of Life Satisfaction on IA

Results of correlation analysis demonstrated significant negative correlations between life satisfaction and IA at each wave (*r* = −0.26 at Wave 1; *r* = −0.27 at Wave 2; *p*s < 0.01). Results of multiple regression analyses revealed that life satisfaction negatively predicted IA concurrently at both waves (Wave 1: *β* = −0.27, *p* < 0.001, Cohen’s *f^2^* = 0.076; Wave 2: *β* = −0.28, *p* < 0.001, Cohen’s *f*^2^ = 0.087, see Table 3). Similar results were found for the longitudinal prediction of life satisfaction on IA (*β* = −0.20, *p* < 0.001, Cohen’s *f^2^* = 0.040, see Table 4). Moreover, life satisfaction also predicted the decrease of Wave 2 IA when Wave 1 IA was controlled (*β* = −0.08, *p* < 0.001, Cohen’s *f^2^* = 0.006, see Table 4).

### 3.4. RQ4: Mediating effect of Life Satisfaction

Mediation analyses via PROCESS replicated the results of multiple regression analyses that PYD attributes positively predicted life satisfaction, and life satisfaction negatively predicted IA, suggesting the potential mediating effect of life satisfaction. Moreover, the indirect effects of all PYD measures on IA via life satisfaction were significant (B ranged between −0.23 and −0.14, *p*s < 0.001, see Table 7).

**Figure 1:**
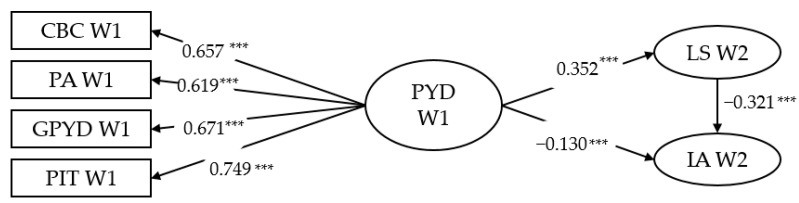
Standardized results of structural equation modeling (SEM) analysis on the relationships among PYD attributes, life satisfaction and Internet addiction. CBC = cognitive-behavioral competence; PA = prosocial attribute; GPYD = general positive youth development; PIT = positive identity; LS = life satisfaction; IA = Internet addiction. W1 = Wave 1; W2 = Wave 2. *** *p* < 0.001.

As PROCESS can only consider PYD predictors each at a time, we further developed a SEM model with latent variables (see Figure 1). This model showed adequate model fit (χ^2^ = 640.472, df = 146, CFI = 0.97, TLI = 0.97, NNFI = 0.97, RMSEA = 0.039 (90% CI = [0.036, 0.042]), SRMR = 0.037) [92]. Table 8 shows the standardized coefficients in this model. Total PYD at Wave 1 significantly and positively predicted Wave 2 life satisfaction (*β* = 0.35, *p <* 0.001). Wave 2 life satisfaction demonstrated a significant and negative prediction on IA (*β* = −0.32, *p <* 0.001). The results of SEM complemented and enriched the PROCESS findings.

## 4. Discussion

Nowadays, adolescents increasingly use the Internet in their daily lives for education, communication, and entertainment purposes. Given the negative impacts of inappropriate Internet use on adolescent development, preventing adolescent IA is an important task for schools, parents, and educational bureaus. This study aims to provide the evidence base for IA prevention through the lens of a strength-based approach. By using two waves of data, this study examined the influence of PYD attributes on IA, and life satisfaction as a mediator in this process among Chinese high school students.

For RQ1, results revealed that all PYD attributes were found to be negatively related to IA at each wave and over time, thus supporting Hypothesis 1a and 1b. The findings were in line with theoretical arguments that cultivating PYD attributes would prevent adolescents from coping with negative life events with IA [20]. Our results echoed previous findings based on both cross-sectional and longitudinal designs in different societies [47,66,93], evincing the protective effects of PYD attributes in preventing adolescent IA.

As to RQ2, results indicated that PYD attributes positively predicted adolescent life satisfaction. The results were in line with previous research showing that adolescents with higher levels of PYD attributes tended to have more positive life experiences and fewer negative life experiences, which subsequently contributed to their general life satisfaction [76]. It is notable that the positive effects of positive identity and general PYD attributes on life satisfaction were particularly powerful and stable. Our results replicated the findings in both Western [75] and Chinese contexts [35,50], supporting the theoretical argument that positive attributes would generally promote human well-being across cultures.

As to RQ3, the present study revealed life satisfaction as a negative predictor of IA, suggesting that adolescents who were satisfied with their lives tended to develop fewer IA symptoms. For adolescents who are dissatisfied with their lives, they may seek pleasure on the Internet, where they can achieve success in online games and communicate comfortably due to the anonymity and privacy of the Internet [94]. Our results were consistent with existing findings in different societies [57,95].

Regarding RQ4, life satisfaction was found as a significant mediator of the relationship between all dimensions and total PYD attributes and IA. The results were in agreement with previous findings revealing positive relations between PYD attributes and life satisfaction and a negative relationship between life satisfaction and problem behaviors [22,79,96]. Our results also expanded previous findings by unraveling the psychological mechanism underlying the relationship between PYD attributes and IA and highlighting the importance of adolescents’ subjective evaluation of their lives in preventing behavioral problems [56,77].

Some observations can be drawn from the results. First, cognitive-behavioral competence was negatively related to IA concurrently and longitudinally. In addition to cognitive behavior approach therapy serving as a treatment and intervention for IA [33], our results suggest the value of developing cognitive-behavioral competence as a prevention tool to restructure cognitions regarding Internet use and modify maladaptive behavior. In addition, our results revealed a positive relationship between cognitive-behavioral competence and life satisfaction, suggesting that adolescents with stronger cognitive competence and behavioral competence tend to feel satisfied with their lives. Although some research has revealed non-significant correlations [62], our results were congruent with previous findings revealing a positive relationship between cognitive-behavioral competence and life satisfaction in the Western [36] and Chinese contexts [35].

Second, positive identity also demonstrated a negative prediction for IA at each wave and in the long run. In addition, results demonstrated that positive identity was a strong and stable predictor of life satisfaction. As searching for identity is one of the significant developmental tasks for adolescents, positive identity construction is vital for adolescent developmental functioning and well-being [97]. According to identity theory, identities give individuals roles in the group and society they are attached to, and related characteristics describing themselves [98]. The verification of identities has been linked to self-worth, self-respect, and positive self-regard, as well as prestige, respect, esteem, recognition, and appreciation from other people [98]. As IA functions as a response to identity confusion, adolescents who experience difficulties in establishing a positive self-image and gaining respect from others in the real world may look for recognition from the online world as compensation. For example, a study revealed that online game players who claimed to feel “more important and more respected in the virtual group” tended to display more IA symptoms [99]. Although it is argued that the Internet can be used as a tool to explore and develop adolescent self-identity, IA may still limit real-world social interactions, slow the formation of positive identity, or even deteriorate self-identity confusion [11,12]. In congruence with previous findings [41], the present findings underscore the protective role of positive identity in IA prevention as it allows adolescents to see themselves as valued and worthwhile in real life [100].

Third, prosocial attributes were also found to negatively predict IA in the present study, although the effect was relatively small compared to other PYD attributes. Social control theory suggests that when adolescents have close social connections or perceive strong social expectations and norms, they feel obligated to behave in non-deviant ways [101]. In other words, adolescents possessing higher levels of prosocial attitudes tend to have stronger perceptions of social norms and commitment to the group they belong to, and thus are more likely to conform to norms and the supervision of adults, including the explicit rules set for Internet use and implicit social expectations. In addition, prosocial attitudes may serve as resources to deal with the perceived reduced accountability due to the anonymity and privacy of the Internet [94]. The findings may also suggest some cultural explanations. It is possible in China, where a Confucian culture and strong collectivist perspective hold sway, a strong level of prosocial attributes would provide protective resources against problem behavior such as IA [102].

Fourth, other general PYD attributes demonstrated negative predictions for IA concurrently and longitudinally. Interestingly, the protective effects of general PYD attributes on IA prevention appeared to be the strongest among the four dimensions of PYD attributes. This suggests that the protective effects of personal strengths, such as emotional quotient, positive social boding, moral competence, spirituality, and resilience, are equally, if not more, important than cognitive-behavioral competence, which has been the main focus in traditional IA intervention [33]. It is possible that adolescents use cognitive-behavioral competence to directly regulate Internet use behavior and alleviate the withdrawal symptoms, while general PYD attributes support the construction of active coping styles and reduce the chance of adopting IA as a coping strategy [103]. This argument is supported by empirical evidence. For example, Wang et al.’s research [104] found that developmental dysfunctions lead adolescents to unhealthy coping styles. 

This research has some theoretical implications. Research on PYD and life satisfaction of adolescents in Chinese societies has only begun in recent years and mainly conducted in Hong Kong [23,79,105]. The present study contributes to the ongoing discussion by exploring this issue among mainland Chinese adolescents. The results generally replicated previous findings in Western [74] and other Chinese societies [5,30,66,89], suggesting that the positive functions of PYD qualities in promoting well-being and preventing health problems might be universal [31].

The present study provides important policy and practical implications for IA prevention. The construction of different dimensions of positive strengths is vital to enhance how adolescents perceive their lives, which subsequently influences their problem behaviors. As life satisfaction is concerned with the perception of one’s life as a whole, it stays considerably stable within a given period [60]. The present study suggests the possibility of enhancing adolescent life satisfaction through promoting related PYD attributes. This invites public efforts for designing and implementing effective PYD programs to increase adolescent life satisfaction and prevent IA. In fact, some PYD programs conducted in Chinese contexts, such as the Project Positive Adolescent Training through Holistic Social Programs (P.A.T.H.S. project) in Hong Kong and Tin Ka Ping P.A.T.H.S. project in mainland China, have been shown to effectively promote adolescent well-being and healthy development [23,106,107]. At the same time, there are views suggesting that different stakeholders see the importance of life skills, but such education is inadequately covered in the formal curriculum [108]. Practically, conducting screening at the beginning of the academic year would help schools identify adolescents at risk, provide early intervention and prevention through enhancing specific personal strengths, make adolescents feel more connected to the real world, and develop interests other than the Internet, which contribute to IA prevention.

Despite the pioneering nature of this study, some limitations should be noted. First, as this short-term longitudinal study was based on two waves of data, the results should be interpreted with caution. Earlier research also suggests that a potential reciprocal relationship may exist in the relationship between life satisfaction and IA [109]. Therefore, a longitudinal design collecting multiple waves of data and testing different relationships among these variables is needed in the future [110]. Second, although the sample size is relatively large, this study was conducted in only four junior schools in three provinces in mainland China. Future work will benefit from including more schools in more cities in China for further generalization. Third, the self-efficacy subscale presented less satisfactory reliability as compared with other primary PYD attributes subscales. The results should be interpreted with caution. Fourth, this study only involved family intactness as a control variable but did not include other family factors such as parental behaviors and parents’ Internet use [10,111]. As a supportive and healthy family environment enables adolescents to feel cared for and respected, it is expected that adolescents growing up in a warm family environment would develop stronger personal strengths than their counterparts. In addition, besides life satisfaction, other psychological processes may affect the relationships between positive adolescent qualities and IA [112]. These factors, such as loneliness, coping styles, and locus of control, along with their influencing pathways on IA, remain under-researched. Besides, given that 99.2% of Internet users in China use a smartphone to access the Internet [1], it would be illuminating to examine whether the protective effects of different dimensions of PYD attributes differ in preventing specific types of Internet use activities, such as online gaming, social media use and mobile apps use. Particularly, the specific choice of Internet activities on online platforms with different orientations (e.g., videos-based versus texts-based) may reflect adolescents’ differentiated motivation and thus should be taken into account in future research [10]. Finally, the data collected were all self-reported by participants. Although self-report measures are commonly used in adolescent research, this method might lead to methodological bias. For PYD measures, data based on the significant others of adolescents, such as parents, teachers, and peers, would add value to the research. For IA measures, automatic tracking data of Internet use can be collected together with self-reported data [113].

## 5. Conclusions

To conclude, the present study generally confirms previous findings on the linkages between PYD attributes, life satisfaction, and IA. Our findings underline the importance of PYD attributes in promoting adolescents’ life satisfaction and IA prevention. Based on the findings, we suggest that effective PYD programs emphasizing a coherent development of PYD attributes have great potential in improving adolescent well-being and healthy development. In view of the lack of longitudinal empirical research studies on adolescent IA, the present study provides empirical evidence for strength-based IA intervention and prevention. The findings could also be used by policymakers and practitioners in allocating resources or developing public health programs aimed to promote adolescent healthy Internet use.

## Figures and Tables

**Table 1:** Reliability of measures.

Measures	Wave 1		Wave 2	
	α	Mean IIC ^1^	α	Mean IIC ^1^
Positive Youth Development (PYD) primary factors				
Bonding	0.82	0.43	0.89	0.57
Resilience	0.83	0.45	0.90	0.61
Social competence	0.84	0.44	0.89	0.55
Recognition for positive behavior	0.78	0.48	0.84	0.58
Emotional competence	0.83	0.45	0.87	0.53
Cognitive competence	0.85	0.48	0.89	0.59
Behavioral competence	0.75	0.38	0.84	0.51
Moral competence	0.76	0.35	0.80	0.41
Self-determination	0.78	0.42	0.84	0.52
Self-efficacy	0.54	0.37	0.59	0.42
Clear and positive identity	0.84	0.43	0.87	0.50
Beliefs in the future	0.75	0.51	0.79	0.56
Prosocial involvement	0.82	0.49	0.88	0.59
Prosocial norms	0.75	0.38	0.81	0.47
Spirituality	0.85	0.46	0.89	0.53
PYD higher-order factors				
Cognitive-behavioral competence	0.91	0.38	0.94	0.49
Prosocial attributes	0.86	0.40	0.90	0.48
General PYD qualities	0.95	0.30	0.96	0.37
Positive identity	0.88	0.44	0.90	0.49
Total PYD	0.97	0.31	0.98	0.38
Life satisfaction	0.81	0.48	0.84	0.53
Internet Addiction	0.77	0.26	0.80	0.29

^1^ IIC = Inter-item correlation.

**Table 2:** Descriptive and correlational analyses.

Measures	Mean	SD	Correlations															
			1	2	3	4	5	6	7	8	9	10	11	12	13	14	15	16
1. Age	13.12	0.81																
2. Gender ^a^			−0.08 ***															
3. Family intactness ^b^			0.02	0.01														
4. W1 IA	2.31	2.35	0.09 ***	−0.17 ***	0.08 ***													
5. W2 IA	2.30	2.44	0.06 **	−0.10 ***	0.05 **	0.46 ***												
6. W1 LS	4.08	1.13	−0.06 **	−0.07 **	−0.07 **	–0.26 **	−0.19 **											
7. W2 LS	4.06	1.13	−0.03	−0.08 **	−0.06 **	–0.16 **	−0.27 **	0.38 **										
8. W1 CBC	4.82	0.74	−0.09 ***	−0.01	−0.05 **	−0.26 ***	−0.17 ***	0.51 **	0.21 **									
9. W1 PA	4.87	0.84	−0.09 ***	0.08 ***	−0.05 *	−0.23 ***	−0.14 ***	0.46 **	0.18 **	0.66 ***								
10. W1 GPYD	4.81	0.71	−0.12 ***	0.01	−0.07 **	−0.31 ***	−0.20 ***	0.60 **	0.27 **	0.83 ***	0.71 ***							
11. W1 PIT	4.53	0.93	−0.11 ***	−0.10 ***	−0.07 **	−0.25 ***	−0.17 ***	0.55 **	0.25 **	0.71 ***	0.59 ***	0.74 ***						
12. W1 TPYD	4.78	0.69	−0.12 ***	−0.01	−0.07 **	−0.31 ***	−0.20 ***	0.61 **	0.27 **	0.90 ***	0.79 ***	0.97 ***	0.83 ***					
13. W2 CBC	4.95	0.78	−0.10 ***	−0.02	−0.02	−0.19 ***	−0.27 ***	0.31 **	0.44 **	0.44 ***	0.35 ***	0.43 ***	0.39 ***	0.46 ***				
14. W2 PA	5.01	0.85	−0.09 ***	0.07 ***	−0.02	−0.20 ***	−0.23 ***	0.28 **	0.44 **	0.34 ***	0.42 ***	0.40 ***	0.32 ***	0.42 ***	0.66 ***			
15. W2 GPYD	4.92	0.76	−0.08 ***	−0.01	−0.03	−0.23 ***	−0.32 ***	0.38 **	0.57 **	0.43 ***	0.38 ***	0.49 ***	0.42 ***	0.50 ***	0.84 ***	0.69 ***		
16. W2 PIT	4.64	0.95	−0.06 **	−0.12 **	−0.04 *	−0.18 ***	−0.27 ***	0.34 **	0.52 **	0.38 ***	0.30 ***	0.41 ***	0.48 ***	0.44 ***	0.72 ***	0.61 ***	0.75 ***	
17. W2 TPYD	4.90	0.73	−0.09 ***	−0.02	−0.03	−0.23 ***	−0.31 ***	0.38 **	0.58 **	0.45 ***	0.41 ***	0.50 ***	0.45 ***	0.51 ***	0.91 ***	0.78 ***	0.97 ***	0.84 ***

*Note.*^a^ 1 = boy, 2 = girl; ^b^ 1 = intact, 2 = non-intact; W1 = Wave 1; W2 = Wave 2; IA = Internet addiction; LS = life satisfaction; CBC = cognitive-behavioral competence; PA = prosocial attribute; GPYD = general positive youth development; PIT = positive identity; TPYD = mean of total positive youth development. * *p* < 0.05; ** *p* < 0.01; *** *p* < 0.001.

**Table 3:** Cross-sectional regression analyses for Internet addiction.

Model	Predictors	Internet Addiction (Wave 1)					Internet Addiction (Wave 2)				
		*β*	*t*	Cohen’s *f^2^*	R^2^ Change	F Change	*β*	*t*	Cohen’s *f^2^*	R^2^ Change	F Change
**1**	Age	0.07	3.77 ***	0.005	0.040	35.54 ***	0.05	2.47 *	0.002	0.014	12.26 ***
	Gender ^a^	−0.16	−8.30 ***	0.026			−0.09	−4.73 ***	0.009		
	Family intactness ^b^	0.08	4.22 ***	0.007			0.05	2.5 *	0.002		
2	CBC	−0.25	−13.33 ***	0.066	0.062	178.20 ***	−0.27	−14.06 ***	0.076	0.070	197.64 ***
	PA	−0.21	−10.98 ***	0.045	0.043	120.51 ***	−0.21	−11.09 ***	0.047	0.045	123.06 ***
	GPYD	−0.30	−16.22 ***	0.097	0.089	263.19 ***	−0.31	−16.69 ***	0.106	0.096	278.60 ***
	PIT	−0.26	−13.89 ***	0.072	0.067	192.96 ***	−0.28	−14.72 ***	0.083	0.077	216.59 ***
	TPYD	−0.30	−16.10 ***	0.096	0.088	259.10 ***	−0.31	−16.66 ***	0.106	0.096	277.43 ***
	LS	−0.27	−14.29 ***	0.076	0.071	204.25 ***	−0.28	−15.05 ***	0.087	0.080	226.65 ***

*Note.* In model 2, control variables were statistically controlled, and predictors were included in the model separately; measures of positive youth development at Wave 1 and Wave 2 were included as predictors to predict internet addiction at Wave 1 and Wave 2, respectively. ^a^ 1 = boy, 2 = girl; ^b^ 1 = intact, 2 = non-intact; CBC = cognitive-behavioral competence; PA = prosocial attribute; GPYD = general positive youth development; PIT = positive identity; TPYD = mean of total positive youth development; LS = life satisfaction. * *p* < 0.05; *** *p* < 0.001.

**Table 4:** Longitudinal regression analyses for Internet addiction.

Model	Predictors	Internet Addiction (Wave 2)					Internet Addiction (Wave 2)				
		*β*	*t*	Cohen’s *f^2^*	R^2^ Change	F Change	*β*	*t*	Cohen’s *f^2^*	R^2^ Change	F Change
1	Age	0.05	2.47 *	0.002	0.014	12.26 ***	0.02	0.90	0.000	0.198	641.68 ***
	Gender ^a^	−0.09	−4.73 ***	0.009			−0.02	−1.08	0.000		
	Family intactness ^b^	0.05	2.50 *	0.002			0.02	0.88	0.000		
	W1 IA						0.45	25.33 ***	0.246		
2	CBC	−0.16	−8.25 ***	0.026	0.025	68.04 ***	−0.05	−2.85 **	0.003	0.002	8.11 **
	PA	−0.14	−6.91 ***	0.018	0.018	47.79 ***	−0.04	−2.42 *	0.002	0.002	5.86 *
	GPYD	−0.20	−10.27 ***	0.040	0.039	105.40 ***	−0.07	−3.80 ***	0.005	0.004	14.47 ***
	PIT	−0.17	−8.83 ***	0.030	0.029	78.01 ***	−0.06	−3.39 **	0.003	0.004	11.49 **
	TPYD	−0.20	−10.08 ***	0.039	0.037	101.51 ***	−0.07	−3.69 ***	0.004	0.004	13.60 ***
	LS	−0.20	−10.18 ***	0.040	0.038	103.63 ***	−0.08	−4.36 ***	0.006	0.006	18.97 ***

*Note.* In model 2, control variables were statistically controlled, and predictors measured at Wave 1 were included in the model separately. ^a^ 1 = boy, 2 = girl; ^b^ 1 = intact, 2 = non-intact; CBC = cognitive behavioral competence; PA = prosocial attribute; GPYD = general positive youth development; PIT = positive identity; TPYD = mean of total positive youth development; LS = life satisfaction. * *p* < 0.05; ** *p* < 0.01; *** *p* < 0.001.

**Table 5:** Cross-sectional regression analyses for life satisfaction.

Model	Predictors	Life Satisfaction (Wave 1)					Life Satisfaction (Wave 2)					
		*β*	*t*	Cohen’s *f^2^*	R^2^ Change	F Change	*β*	*t*	Cohen’s *f^2^*	R^2^ Change	F Change	
1	Age	−0.07	−3.68 ***	0.005	0.014	12.43 ***	−0.03	−1.62	0.001	0.011	9.65 ***	
	Gender ^a^	−0.07	−3.73 ***	0.005			−0.09	−4.42 ***	0.008			
	Family intactness ^b^	−0.07	−3.34 ***	0.004			−0.05	−2.72 **	0.003			
2	CBC	0.51	29.82 ***	0.338	0.253	889.31 ***	0.44	24.86 ***	0.237	0.192	618.23 ***	
	PA	0.48	27.55 ***	0.289	0.224	758.81 ***	0.44	24.71 ***	0.234	0.190	610.59 ***	
	GPYD	0.60	38.21 ***	0.553	0.356	1459.64 ***	0.58	35.88 ***	0.491	0.329	1287.62 ***	
	PIT	0.55	33.44 ***	0.425	0.298	1118.14 ***	0.56	34.09 ***	0.444	0.308	1162.41 ***	
	TPYD	0.61	39.38 ***	0.587	0.370	1550.97 ***	0.58	36.05 ***	0.495	0.331	1299.26 ***	

*Note.* In model 2, control variables were statistically controlled, and predictors were included in the model separately; measures of positive youth development at Wave 1 and Wave 2 were included as predictors to predict internet addiction at Wave 1 and Wave 2, respectively. ^a^ 1 = boy, 2 = girl; ^b^ 1 = intact, 2 = non-intact; CBC = cognitive-behavioral competence; PA = prosocial attribute; GPYD = general positive youth development; PIT = positive identity; TPYD = mean of total positive youth development. ** *p* < 0.01; *** *p* < 0.001.

**Table 6:** Longitudinal regression analyses for life satisfaction.

Model	Predictors	Life Satisfaction (Wave 2)					Life Satisfaction (Wave 2)				
		*β*	*t*	Cohen’s *f^2^*	R^2^ Change	F Change	*β*	*t*	Cohen’s *f^2^*	R^2^ Change	F Change
1	Age	−0.03	−1.62	0.001	0.011	9.65 ***	0.00	−0.19	0.000	0.137	412.76 ***
	Gender ^a^	−0.09	−4.42 ***	0.008			−0.06	−3.32 ***	0.004		
	Family intactness ^b^	−0.05	−2.72 **	0.003			−0.03	−1.58	0.001		
	W1 LS						0.37	20.32 ***	0.159		
2	CBC	0.21	10.64 ***	0.043	0.042	113.12 ***	0.02	0.93	0.000	0.000	0.87
	PA	0.18	9.33 ***	0.033	0.032	87.08 ***	0.00	−0.08	0.000	0.000	0.01
	GPYD	0.27	14.38 ***	0.079	0.073	206.83 ***	0.07	3.11 **	0.003	0.003	9.67 **
	PIT	0.24	12.56 ***	0.061	0.057	157.83 ***	0.05	2.14 *	0.002	0.002	4.6 *
	TPYD	0.27	13.99 ***	0.075	0.070	195.86 ***	0.06	2.37 *	0.002	0.002	5.64 *

*Note.* In model 2, control variables were statistically controlled, and predictors measured at Wave 1 were included in the model separately. ^a^ 1 = boy, 2 = girl; ^b^ 1 = intact, 2 = non-intact; CBC = cognitive behavioral competence; PA = prosocial attribute; GPYD = general positive youth development; PIT = positive identity; TPYD = mean of total positive youth development; LS = life satisfaction. * *p* < 0.05; ** *p* < 0.01; *** *p* < 0.001.

**Table 7:** Longitudinal mediating effect analyses of life satisfaction at Wave 2 (the mediator) for the effect of PYD measures at Wave 1 on Internet addiction at Wave 2.

Regression Models Summary	Independent Variables (IV) at Wave 1														
	CBC			PA			PIT			GPYD			TPYD		
	*B*	*SE*	*t*	*B*	*SE*	*t*	*B*	*SE*	*t*	*B*	*SE*	*t*	*B*	*SE*	*t*
Total effect of IV on DV	−0.53	0.06	−8.28 ***	−0.40	0.06	−6.96 ***	−0.45	0.05	−8.91 ***	−0.69	0.07	−10.29 ***	−0.69	0.07	−10.12 ***
IV to Mediator	0.31	0.03	10.53 ***	0.25	0.03	9.44 ***	0.29	0.02	12.47 ***	0.44	0.03	14.34 ***	0.43	0.03	13.95 ***
Mediator to DV	−0.56	0.04	−13.53 ***	−0.57	0.04	−13.92 ***	−0.55	0.04	−13.20 ***	−0.53	0.04	−12.61 ***	−0.53	0.04	−12.71 ***
Direct effect of IV on DV	−0.36	0.06	−5.65 ***	−0.26	0.06	−4.55 ***	−0.30	0.05	−5.80 ***	−0.46	0.07	−6.78 ***	−0.46	0.07	−6.70 ***
Mediating effect	PE	Bootstrapping (BC 95% CI)		PE	Bootstrapping (BC 95% CI)		PE	Bootstrapping (BC 95% CI)		PE	Bootstrapping (BC 95% CI)		PE	Bootstrapping (BC 95% CI)	
		Lower	Upper		Lower	Upper		Lower	Upper		Lower	Upper		Lower	Upper
	−0.17 ***	−0.22	−0.13	−0.14 ***	−0.18	−0.10	−0.16 ***	−0.20	−0.12	−0.23 ***	−0.28	−0.18	−0.23 ***	−0.29	−0.18

*Note*. In all analyses, control variables were statistically controlled. CBC = Cognitive-behavioral competence; PA = Prosocial attributes; PIT = Positive identity; GPYD = General positive youth development qualities; TPYD = Total positive youth development qualities; IV = Independent variable; DV = Dependent variable; PE= Point estimate; BC = Bias corrected; CI = Confidence interval. *** *p* < 0.001.

**Table 8:** Results of SEM analysis.

Paths		*B*	*β*	*SE*	z-Value	*p* Value
W1 PYD	→W2 Life satisfaction	0.565	0.352	0.041	13.661	0.000
W1 PYD	→W2 IA	−0.044	−0.131	0.009	−4.641	0.000
W2 Life satisfaction	→W2 IA	−0.067	−0.321	0.007	−10.317	0.000
					Bootstrapping 95% CI	
		*B*	*β*	*SE*	Lower	Upper
Indirect effect of W1 PYD on W2 IA through W2 life satisfaction		−0.025	−0.046	0.006	−0.053	−0.019
Direct effect of W1 PYD on W2 IA		−0.044	−0.131	0.009	−0.061	−0.024

*Note.* CBC = cognitive-behavioral competence; PA = prosocial attribute; GPYD = general positive youth development; PIT = positive identity; TPYD = mean of total positive youth development.

## Data Availability

The data presented in this study are available on request from the corresponding author.
